# The Interface between Gd and Monolayer MoS_2_: A First-Principles Study

**DOI:** 10.1038/srep07368

**Published:** 2014-12-08

**Authors:** Xuejing Zhang, Wenbo Mi, Xiaocha Wang, Yingchun Cheng, Udo Schwingenschlögl

**Affiliations:** 1 Tianjin Key Laboratory of Low Dimensional Materials Physics and Preparation Technology, Faculty of Science, Tianjin University, Tianjin 300072, China; 2 Tianjin Key Laboratory of Film Electronic & Communicate Devices, School of Electronics Information Engineering, Tianjin University of Technology, Tianjin 300191, China; 3 PSE Division, KAUST, Thuwal 23955-6900, Kingdom of Saudi Arabia

## Abstract

We analyze the electronic structure of interfaces between two-, four- and six-layer Gd(0001) and monolayer MoS_2_ by first-principles calculations. Strong chemical bonds shift the Fermi energy of MoS_2_ upwards into the conduction band. At the surface and interface the Gd *f* states shift to lower energy and new surface/interface Gd *d* states appear at the Fermi energy, which are strongly hybridized with the Mo 4*d* states and thus lead to a high spin-polarization (ferromagnetically ordered Mo magnetic moments of 0.15 μ_B_). Gd therefore is an interesting candidate for spin injection into monolayer MoS_2_.

Monolayer transition metal dichalcogenides, especially MoS_2_, have promising prospects in many fields due to their exotic electronic, optical, chemical and thermal properties^1^,^2^,^3^,^4^. Unlike gapless graphene, monolayer MoS_2_ has a direct optical band gap of 1.8 eV^5^,^6^, which is key for field effect transistors, photodetectors and electroluminescent devices^7^,^8^,^9^. On the other hand, the low electron mobility hampers high performance applications. Interfaces often are more crucial to nanoelectronics than the involved semiconductors themselves^10^,^11^. Based on density functional theory, Gan *et al.* have shown that the chemical bonds formed at the MoS_2_/TiC interface result in conductive MoS_2_^12^ and Feng *et al.* have predicted that the interfacial hybridization in Fe_4_N/MoS_2_ superlattices results in magnetic MoS_2_^13^. Pb, Au and Ag contacts to monolayer MoS_2_ can be used to realize good electron injection^14^. Popov *et al.*, on the other hand, have observed that Au is rather inefficient for electron injection and have proposed Ti as alternative electrode material^15^. Moreover, Chen *et al.* have demonstrated a *n*-type Schottky-barrier for the contact between monolayer MoS_2_ and Ir(111), Pd(111), or Ru(0001)^16^.

Clearly, interfaces between semiconductors and metals are critical for future electronic devices based on this new class of materials. In particular, injection of spin-polarized charge from ferromagnets may have a significant technological impact in the area of spintronics. Gd is one of the four room-temperature ferromagnetic metals (Curie temperature 293 K; the others being Fe, Co, and Ni). A significant enhancement of the Curie temperature by 29% has been found experimentally at the Gd(0001) surface^17^. In contrast to transition metals, the ferromagnetic order generated by the localized Gd 4*f* electrons also polarizes the conduction electrons (Gd 5*d* and 6*s*), leading to a large magnetic moment of 7.63 μ_B_/Gd^18^. Moreover, Gd crystallizes in the hcp structure with less than 1% lattice mismatch to MoS_2_ and has a low work function of 3.1 eV^19^, thus being able to efficiently inject electrons into the conduction band of MoS_2_. For these reasons, we investigate in the present work, the electronic structure of interfaces between two-, four- and six-layer Gd(0001) and monolayer MoS_2_ by density functional theory, demonstrating great potential for spin injection.

## Methods

Our first-principles calculations are performed using the projector-augmented wave method as implemented in the Vienna *Ab-initio* Simulation Package^20^,^21^. For the exchange-correlation potential we use the generalized gradient approximation (GGA) of Perdew, Burke and Ernzerhof^22^. Due to strong on-site Coulomb repulsion of the localized Gd 4*f* electrons, the rotationally invariant GGA+*U* method is employed with *U* = 7.7 eV and *J* = 0.7 eV^23^. The two-layer Gd/MoS_2_ interface is also studied taking into account the spin-orbit coupling (GGA+SOC). In all calculations the Gd 5*s*, 5*p*, 6*s*, 5*d*, and 4*f* orbitals are treated as valence states, a *Γ*-centered 4 × 4 × 1 *k*-grid is employed, and the plane wave energy cutoff is set to 600 eV. Furthermore, the convergence criterium for the total energy is chosen to be 10^−5^ eV. The surface unit cell of Gd(0001) has *p*(3 × 3) periodicity with experimental lattice constant 10.89 Å^24^, while the surface unit cell of monolayer MoS_2_ has 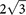 × 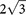 R30° periodicity with experimental lattice constant 10.98 Å^25^. Thus, the mismatch amounts only to 0.83%. The cell volume is relaxed and the ionic positions are optimized, using the conjugated gradient method, until the Hellmann-Feynmann forces on each atom are reduced to less than 0.01 eV/Å. A 15 Å thick vacuum layer ensures decoupling in the slab geometry. Because of strong chemical bonding between Gd(0001) and monolayer MoS_2_, van der Waals forces are not taken into account.

## Results and Discussion

**Figure 1:**
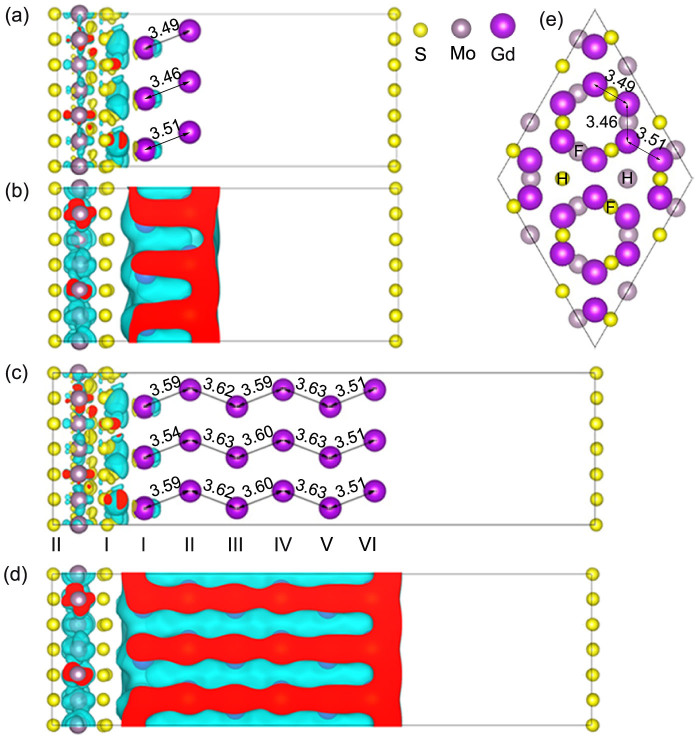
(a, c) Side view of the charge density difference due to the interaction at the two- and six-layer Gd/MoS_2_ interfaces. The cyan and yellow isosurfaces (±0.003 e/Å^3^) represent accumulation and depletion of electrons, respectively. (b, d) Side view of the spin density difference for the two- and six-layer Gd/MoS_2_ interfaces. The isosurface value is 0.002 e/Å^3^. Red color indicates cuts through the isosurface. (e) Top view of the optimized two-layer Gd/MoS_2_ interface.

Bulk MoS_2_ has a layered 2*H* structure with space group *P*6_3_*mmc* (*D*_6*h*_ point group). The trigonal prismatic coordination of the bulk is maintained in monolayer MoS_2_, whereas the symmetry is reduced to 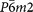 (*D*_3*h*_ point group) due to a loss of inversion symmetry. Gd crystallizes in a hcp structure with space group *P*6_3_*mmc*. The optimized geometries of the interfaces between two- and six-layer Gd and monolayer MoS_2_ are shown in Fig. 1. The results for the interface between four-layer Gd and monolayer MoS_2_ turn out to be very similar to those of the six-layer system and thus are not further discussed in the following. According to Fig. 1(e), three S and Mo atoms in each layer sit above the hexagonal (H) hollow sites and nine S and Mo atoms are located above face-centered (F) hollow sites. The optimized lattice constants of Gd and MoS_2_ are 3.65 and 3.18 Å, respectively, whereas for both the two- and six-layer Gd/MoS_2_ interfaces we obtain 11.03 Å (3.68 and 3.18 Å for Gd and MoS_2_). This means that there is almost no strain. In order to quantify the interaction strength between Gd and MoS_2_, we calculate the binding energy *E*_B_ = *E*_I_ − *E*_M_ − *E*_Gd_, where *E*_I_, *E*_M_, and *E*_Gd_ represent the total energies of the Gd/MoS_2_ interface, monolayer MoS_2_, and the Gd slab, respectively. We obtain per surface Gd atom values of −0.62 and −0.64 eV for the two- and six-layer Gd/MoS_2_ interfaces, reflecting substantial bonding. The distance between the S_I,F_ (the first index refers to the layer and the second to the site) and Mo_F_ atoms, respectively, and their nearest Gd neighbors is 2.77 and 4.23 Å (2.76 and 4.21 Å) in the two-layer (six-layer) Gd/MoS_2_ interface, whereas the corresponding distance for the S_I-H_ and Mo_H_ atoms is larger, namely, 3.17 and 4.69 Å (3.14 and 4.67 Å).

**Figure 2:**
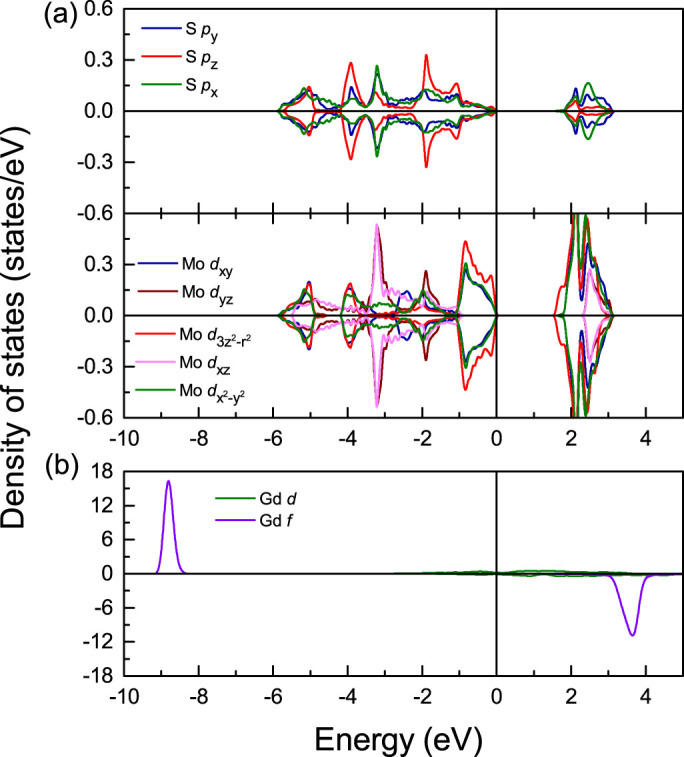
DOS of (a) the Mo and S atoms in pristine monolayer MoS_2_ and (b) the Gd atoms in bulk Gd.

**Figure 3:**
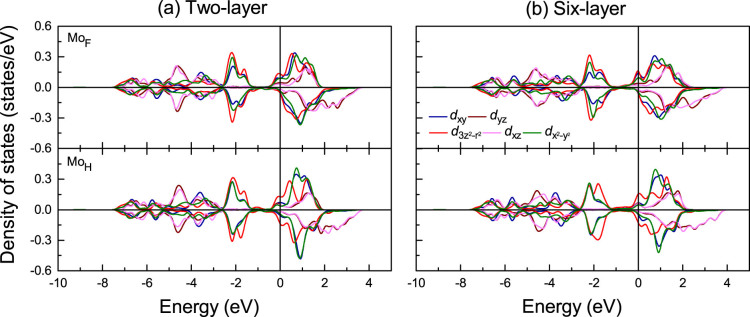
DOS of the Mo atoms at the F and H sites for the two- and six-layer Gd/MoS_2_ interfaces.

**Figure 4:**
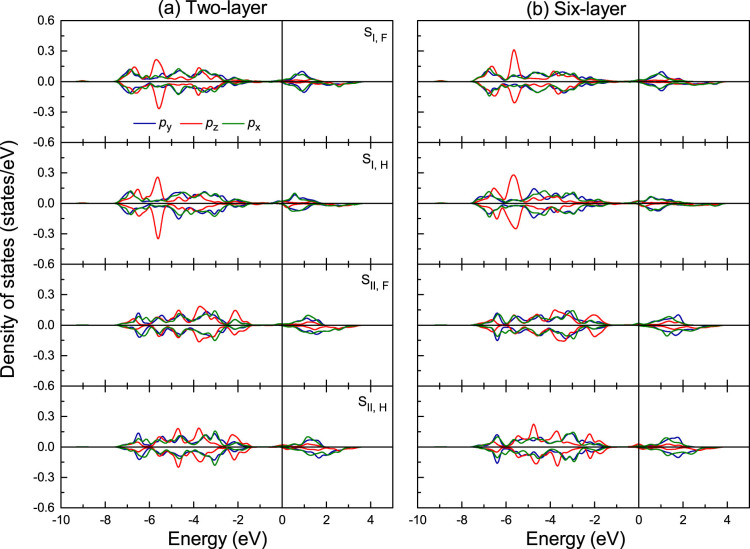
DOS of the S atoms at the F and H sites for the two- and six-layer Gd/MoS_2_ interfaces.

The density of states (DOS) of pristine monolayer MoS_2_ is addressed in Fig. 2(a). The crystal-field splitting of the Mo 4*d* states in the trigonal prismatic environment of the S atoms is visible. Hybridization between the Mo 4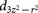, *d_xy_*, 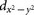 and S 3*p* states at the conduction and valence band edges is consistent with previous results^26^. Figures 3 and 4 give the DOSs obtained for the two- and six-layer Gd/MoS_2_ interfaces. The majority spin Mo_F_ states at the Fermi energy (*E*_F_) display high 4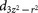, *d_xy_*, and 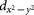 DOSs with *d_yz_* and *d_xz_* admixtures, while the minority spin DOSs are small. The majority spin Mo_H_ DOS at *E_F_* is slightly larger than the minority spin DOS (mainly 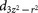 states, followed by *d_xy_*, 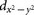 and *d_yz_*, *d_xz_* states). Furthermore, the broader peaks in Fig. 3(a) as compared to Fig. 3(b) reflect more dispersive bands in the two-layer Gd/MoS_2_ interface. To illustrate the charge transfer, we show the charge density difference between the Gd/MoS_2_ interfaces and the sum of the isolated Gd and MoS_2_ subsystems in Figs. 1(a) and (c). Charge accumulates at the Mo atoms and in the Gd-S bond region. The Mo excess electrons populate majority spin states, resulting in enhanced Mo 4*d* magnetic moments. Figure 4 demonstrates that for S_II_ the *p_z_* DOS is larger than the *p_x_* and *p_y_* DOSs, similar to pristine monolayer MoS_2_, at the valence band edge, while for S_I_ mainly the *p*_x_ and *p_y_* orbitals contribute. This means that the Gd-S_II_ interaction is weak (large distance). Due to hybridization with the Gd 5*d* states (details later), some S *p* states show up at *E_F_*, especially majority spin states, which leads to a tiny S magnetic moment (0.01 μ_B_). It is worth noting that, due to the nonmagnetic nature of MoS_2_, we have set the initial magnetic moments of S and Mo to zero in all calculations. The spatial extension of the spin density in MoS_2_ induced by the contact to Gd is shown in Figs. 1(b) and (d). It mainly extends into the Mo region and is small for S (large change of the Mo DOS). We find that the Mo magnetic moments order ferromagnetically. The shorter Mo_F_-Gd distance as compared to the Mo_H_-Gd distance enhances the interaction so that Mo_F _has a larger magnetic moment of about 0.12 and 0.15 μ_B_ (Mo_H_: 0.07 and 0.10 μ_B_) in the two- and six-layer Gd/MoS_2_ interfaces, respectively.

**Figure 5:**
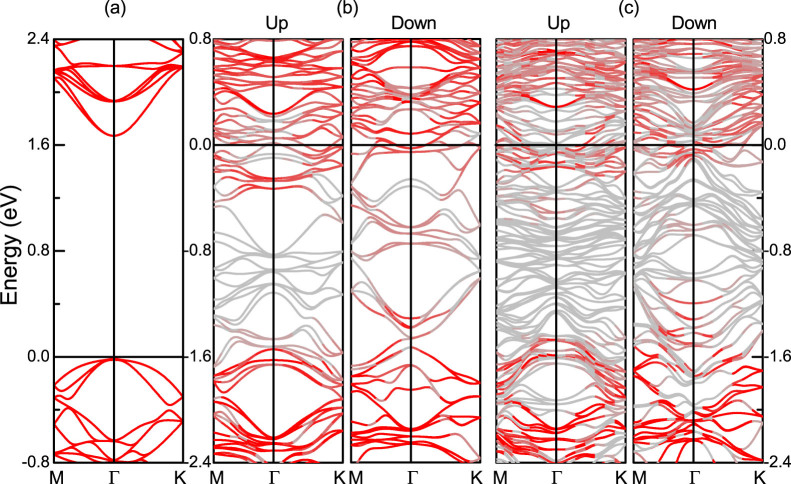
Band structure of (a) pristine monolayer MoS_2_ and (b, c) the two- and six-layer MoS_2_ interfaces. *E_F_* = 0 eV. The red lines correspond to the bands of monolayer MoS_2_.

**Figure 6:**
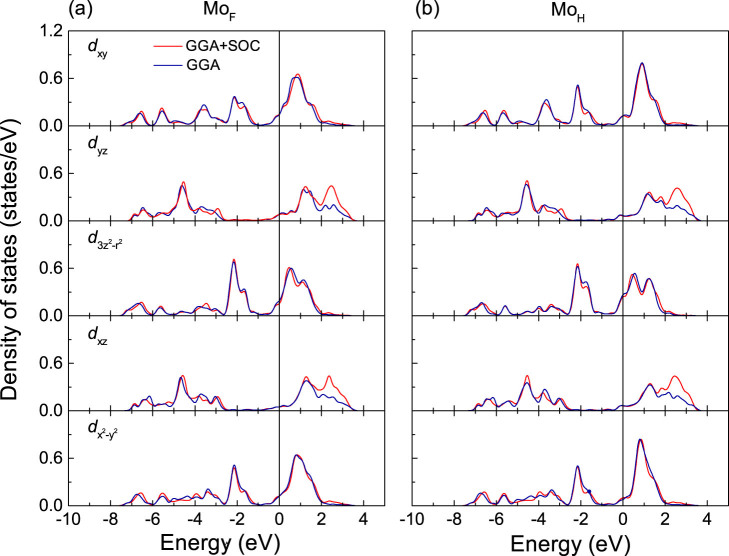
DOS (sum of both spin channels) of the Mo atoms at the F and H sites for the two-layer Gd/MoS_2_ interface: Comparison between the GGA and GGA+SOC methods. *E_F_* = 0 eV.

Figure 5(a) shows a band gap of 1.6 eV for pristine monolayer MoS_2_, consistent with previous GGA calculations^27^. In the combined systems, although the bands of MoS_2_ hybridize with those of Gd they can still be identified, see the red color in Figs. 5(b) and (c). We find *E_F_* 0.34 and 0.51 eV, respectively, above the conduction band edge for the majority and minority spin bands, making MoS_2_ display a metallic character. Figs. 6(a) and (b) show the DOS for Mo_F_ and Mo_H_ in the two-layer Gd/MoS_2_ interface as obtained by GGA+SOC in comparison to simple GGA. We find that the SOC has almost no influence, except for a slight reduction of the 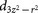 DOS.

The distance between nearest neighbor atoms is 3.60 Å in bulk Gd, while the distances of nearest neighbor atoms in the interface and surface Gd layers, see Figs. 1(a, e) and (c), respectively, are smaller. The very short distance between layers V and VI in the six-layer Gd/MoS_2_ interface points to a substantial surface relaxation. On the other hand, the distances in the subsurface, see Fig. 1(c), are larger than the bulk value. This variation is consistent with a contraction of the surface layer by 0.085–0.115 Å (~3–4%) and an expansion of the subsurface layer by 0.050–0.075 Å (~1.5–2.5%) as measured by low-energy electron diffraction^28^,^29^.

**Figure 7:**
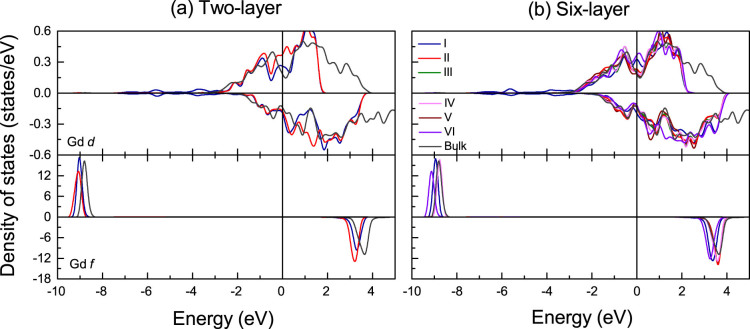
DOS of the Gd atoms in the different atomic layers of the two- and six-layer Gd/MoS_2_ interfaces. *E_F_* = 0 eV.

In bulk Gd the unoccupied *f* states are located 3.6 eV above *E_F_* and the occupied *f* states 8.8 eV below *E_F_*, see Fig. 2(b), reflecting an exchange spin splitting of 12.4 eV. This value agrees with results of the full potential linear augmented plane wave method^30^ and is close to the experimental value of 12 eV^31^. The fact that the Gd magnetic moment (7.43 μ_B_) exceeds 7 μ_B_ suggests an induced polarization of other orbitals. We find magnetic moments of 0.02, 0.03 and 0.40 μ_B_ for the Gd *s*, *p*, and *d* states, which can be explained by the *s*-*f* exchange model^32^. The total Gd magnetic moments are enhanced in the surface Gd layers by 0.9% and 1.9% for the two- and six-layer Gd/MoS_2_ interfaces, respectively, and reduced by 0.9% and 0.5% in the interface Gd layers. The magnetic moments of the different layers given in Table 1 show no effect for the *f* states; all changes are carried by the *d* states. The Gd *d* DOS in Fig. 7, in contrast to the bulk, shows majority spin states from −0.5 to 0.4 eV (from −0.2 to 0.2 eV) for the two-layer (six-layer) Gd/MoS_2_ interface for the surface^33^,^34^,^35^,^36^,^37^ (more pronounced) and interface Gd atoms. Moreover, a strong hybridization between the Gd *d*, Mo *d* and S *p* states appears near *E_F_* (see Figs. 3, 4 and 7). The majority and minority spin Gd *d* DOSs at *E_F_* are different because of an enhancement in the surface and a reduction in the interface Gd layers. In the other Gd layers the Gd magnetic moments are close to the bulk value of 7.43 μ_B_. The Gd *f* DOSs of layers I and II, respectively, show downward shifts of 0.2 and 0.3 eV for the majority spin states and of 0.3 and 0.4 eV for the minority spin states, relative to the bulk Gd *f* states, where the different amplitude is due to the interaction with MoS_2_ in layer I. The same is found for the I and VI layers in the six-layer Gd/MoS_2_ interface, which is consistent with inverse photoemission spectroscopy^37^, while for the II, III, IV, and V layers the shifts are very small. Gd core-level shifts can be attributed to the different chemical environments of the surface and interface atoms, thus being small in other layers.

## Conclusion

We have investigated the geometry, electronic structure, and magnetism at the interface between Gd(0001) and monolayer MoS_2_. Strong chemical bonds are formed and seriously modify the electronic states of MoS_2_, especially at *E_F_*. Interaction with the Gd *d* states shifts *E_F_* into the conduction band and makes MoS_2_ metallic. Large magnetic moments appear on the Mo atoms. Moreover, distinct surface/interface Gd *d* states are formed at *E_F_* and a clear downward shift of the Gd *f* states is observed for both the surface and interface, whereas the Gd magnetic moments are enhanced at the surface but reduced at the interface.

## Author Contributions

X.Z. and W.M. designed the outline of the manuscript and wrote the main text. U.S. gave many good suggestions and contributed detailed discussions and revisions. X.W. and Y.C. contributed detailed discussions and revisions. All authors reviewed the manuscript.

## Figures and Tables

**Table 1:** Gd 5*d* and 4*f* magnetic moments (μ_B_) in each layer of the two- and six-layer Gd/MoS_2_ interfaces, as compared to bulk Gd

System	Layer	*d*	*f*
Bulk		0.4	7.0
Two-layer	I	0.3	7.0
	II	0.5	7.0
Six-layer	I	0.4	7.0
	II	0.4	7.0
	III	0.4	7.0
	IV	0.4	7.0
	V	0.4	7.0
	VI	0.5	7.0
